# Self-Assemblies of Single-Walled Carbon Nanotubes through Tunable Tethering of Pyrenes by Dextrin for Rapidly Chiral Sensing

**DOI:** 10.1155/2011/862692

**Published:** 2011-07-28

**Authors:** Wei-Li Wei, Qiushui Chen, Haifang Li, Jin-Ming Lin

**Affiliations:** ^1^State Key Laboratory of Rare Earth Resource Utilization, Changchun Institute of Applied Chemistry, Chinese Academy of Sciences, Changchun, Jilin 130022, China; ^2^Beijing Key Laboratory for Analytical Methods and Instrumentation, Department of Chemistry, Tsinghua University, Beijing 100084, China

## Abstract

Pyrene-modified dextrin (Py-Dex) was synthesized via the Schiff base reaction between reducing end of dextrins and 1-aminopyrene, and then self-assemblies of single-walled carbon nanotubes (SWNTs) were fabricated through the tunable tethering of pyrene to SWNTs by dextrin chains. The Py-Dex-SWNTs assemblies were found to be significantly water-soluble because of the synergistic effect of dextrin chains and pyrene moieties. Py-Dex and Py-Dex-SWNTs were adequately characterized by NMR, UV-vis, fluorescence spectroscopy, Raman spectroscopy, matrix-assisted laser desorption/ionization-time of flight mass spectroscopy, and transmission electron microscopy. The tethering effect of dextrin toward pyrene moieties was clearly revealed and was found to be tunable by adjusting the length of dextrin chains. The fluorescence of pyrene moieties was sufficiently quenched by SWNTs with the support of dextrin chains. Furthermore, the Py-Dex-SWNTs assemblies were used for chiral selective sensing by introducing cyclodextrins as chiral binding sites. The rapid chiral sensing was successfully tested for different enantiomers.

## 1. Introduction

Chirality plays an important role in various fields such as pharmaceuticals and biotechnology. The booming development of chirality researches demands rapid and sensitive analytical tools for chiral assays. During the past several decades, numerous studies have been focused on chiral analysis resulting in various techniques including various types of chromatography [1], capillary electrophoresis [2, 3], and chiral sensors [4]. Amongst those methods, the use of fluorescence has attracted much interest because it can offer the advantages of real-time analysis, high sensitivity, multiple sensing modes, and remote detection capabilities [5]. Chiral selective fluorescent methods are especially useful for the rapid and high-throughput assay of the enantiomeric composition of chiral substrates [6]. Generally, such a fluorescent method composed of a fluorophore and a chiral binding site. The chirality of the binding site could be achieved by introducing chiral selectors into the binding site. Significantly, some chiral cyclic compounds such as crown ethers, calixarenes, and cyclodextrins (CDs) have been found to be versatile chiral selectors through chiral selective inclusion. Thus, these compounds could be fabricated into fluorescence sensors for chiral recognition. 

On the other hand, as a result of the extraordinary chemical, electronic, and mechanical properties of single-walled carbon nanotubes (SWNTs), SWNTs-mediated self-assemblies are becoming attractive with the potential for applications ranging from photo-electrochemical devices [7] to biosensors [8]. Theoretical [9] and experimental [10] studies have demonstrated that SWNTs could act as quenchers for fluorophores. This discovery provides a base for fluorescent sensing. Recently, self-assembly of SWNTs and single-stranded DNA (ssDNA) has been applied to the probing of biomolecules via fluorescence restoration [11]. These applications led us to explore the design and construction of SWNTs-mediated self-assemblies to extend the applications of SWNTs in molecular sensing and particularly in chiral selective sensing, which has not been reported to the best of our knowledge. Linear polymers such as ssDNA and amyloses, which are *α*-(1,4)-linked dextrose chains, have been found that they could helically wrap SWNTs [12]. However, the ssDNA is usually expensive and unavailable. Although the amylose is readily available, it is hardly water soluble. Therefore, discovery of a water soluble and readily available “molecular rope” is important to achieve our strategy. The short chain analogues of amylose, namely, dextrin, can satisfy the two requirements. Nevertheless, the dextrin alone was found to be a poor SWNTs modifier [13], because it could not effectively disperse SWNTs in water. Hence, pyrene ring was chosen as fluorophore, considering that pyrene rings could additionally support the dispersion of SWNTs [14]. Generally, pyrene rings were introduced into polymers for the better modification of SWNTs, but the combination of pyrene and dextrin was not reported, as far as we know.

**Scheme 1:**
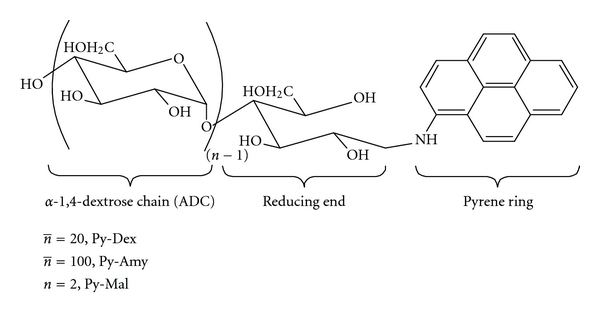
The construction of 1-aminopyrene-modified dextrin.

In this work, we proposed a strategy to noncovalently tether a fluorophore to SWNTs through the wrapping of a polymer chain (namely, “molecular rope”) around the SWNTs. With tethering, the controllable attachment between the fluorophore and SWNTs would be achieved. The construction of self-assembly based on 1-aminopyrene-modified dextrin (Py-Dex) (Scheme 1) and SWNTs is described. The 1-aminopyrene-modified amylose (Py-Amy) was also provided for the comparison of the tethering effect of different dextrose chain length. Their applications in chiral selective sensing were further investigated in detail. The cooperation between pyrene and dextrin extraordinarily improved the water solubility of SWNTs. Significantly, the homogeneous aqueous solutions of the self-assembly of Py-Dex and SWNTs (Py-Dex-SWNTs) have been shown as stable for more than two weeks, and the employed SWNTs also played a crucial role in chiral selective sensing, which will serve us to further comprehend the application of SWNTs in the molecular recognition studies.

## 2. Experimental Section

### 2.1. Materials

Dextrin (CAS 9004-53-9) was purchased from Sigma-Aldrich Co. (MO, USA) and used without any purification. Amylose (average DP = 100) was purchased from Sinopharm Chemical Reagent Co. (Shanghai, China). *α*-cyclodextrin (*α*-CD), *β*-cyclodextrin (*β*-CD), and *γ*-cyclodextrin (*γ*-CD) of analytical grade were all purchased from Maxdragon (G.Z.) BioChem Ltd. (Guangzhou, China). 1-Aminopyrene (with >98% purity) was purchased from Acros Organic (Geel, Belgium). Single-walled carbon nanotubes (SWNTs, purity >90 wt%, diameter: 1-2 nm, length: ca. 20 *μ*m) produced by high-pressure CO conversion (HiPco) were obtained from Chengdu Organic Chemicals Co. Ltd., Chinese Academy of Sciences, China. *R*-, *S*-2-phenylglycinol, *R*-, *S*-*α*-methylbenzylamine, and D-, L-phenylalanine (all with >99% purity) were purchased from J&K Scientific Ltd. (Beijing, China). Ultrapure water was used in all the experiments. Other reagents were all of analytical grade.

### 2.2. Instrumentations

UV-vis spectra were recorded in a conventional quartz cell (light path 10 mm) on a Shimadzu UV-2401PC spectrophotometer (Shimadzu, Kyoto, Japan). The fluorescence measurements were carried out on a Hitachi F-7000 spectrophotometer (Tokyo, Japan). ^1^H NMR spectra were recorded in DMSO-D6 solution at 25°C on a 600 MHz pulsed Fourier Transform NMR spectrometer (JOEL JNM-ECA600, Tokyo, Japan). Raman spectra were recorded on Renishaw RM1000 research laser Raman microscope with excitation of 633 nm. Transmission electron microscopy (TEM) experiments were performed using a Hitachi H-800 electron microscope operating at 200 kV. For visualization by TEM, samples were prepared by dropping a solution of production on a copper grid. Quadrupole time-of-flight (Q-TOF) mass spectra were recorded on Bruker Daltonics micrOTOF-Q II. Matrix-assisted laser desorption/ionization-time of flight (MALDI-TOF) mass spectra were carried out with an autoflex workstation (Bruker Daltonics, Germany) equipped with a nitrogen laser of 337 nm. The mass spectrometer was selected for positive ions. After a delayed extraction of 100 ns, the ions were accelerated to a kinetic energy of 20 kV. Hereafter, the ions were detected in the reflector mode. The lowest power required to obtain good spectra was used. The mixture of 0.5 *μ*L sample and 0.5 *μ*L of the matrix was dried on a sample plate. The matrix solution was composed of 0.2 M 2,5-dihydroxybenzonic acid and 0.06 M 1-hydroxy isoquinoline in water/acetonitrile (1 : 1, v/v) mixture containing 0.1% trifluoroacetic acid.

### 2.3. Preparation of Py-Dex

The preparation of Py-Dex was based on the reports of Novotny's group [15–17]. Dextrin (70 mg) was dissolved in a 15 mL volume of 1 : 1 mixture of 40 mM aqueous phosphoric acid/DMSO with sonication. After added 1-aminopyrene (20 mg), the system was stirred at 85°C for 45 minutes under a nitrogen atmosphere and then cooled to room temperature. Then, sodium cyanoborohydride (NaCNBH_3_) was added to a final concentration of 0.2 M. Subsequently, the mixture was maintained at 85°C for 10 hours. Purification procedures were carried out. Water was firstly removed from the reacted mixture by vacuum distillation and the inorganic salts precipitated out of the mixture following the removal of water. The product of Py-Dex was precipitated out from the supernatant by acetone. The precipitate was sufficiently washed with acetone until no 1-aminopyrene was detected in the washed acetone using fluorescence spectrometer and dried under nitrogen to give purified Py-Dex. UV/Vis (water): *λ*_max_ = 357, 283, and 242 nm; fluorescence (water): excitation/emission = 360/441 nm; ^ 1^H NMR (600 MHz, DMSO-D6, 25°C) *δ*  = 8.2665–7.6641 (9H, protons of pyrene moieties), 5.0519–3.0260 (protons of the glucose unit and ring opened reducing end). MALDI-TOF MS (*m/z*): [M+H]^+^: 867.229 (DP4), 1029.375 (DP5), 1191.497 (DP6), 1353.564 (DP7), 1515.603 (DP8), 1677.614 (DP9), 1839.585 (DP10), 2001.512 (DP11), 2163.431 (DP12), 2325.343 (DP13), 2487.299 (DP14), 2649.139 (DP15), 2810.847 (DP16), 2974.266 (DP17), 3135.671 (DP18); [M+Na]^+^: 890.264 (DP4), 1052.389 (DP5), 1214.493 (DP6), 1376.569 (DP7), 1538.607 (DP8), 1700.594 (DP9), 1862.567 (DP10), 2024.499 (DP11), 2186.415 (DP12), 2348.326 (DP13), 2510.213 (DP14), 2672.07 (DP15), 2833.992 (DP16), 2995.765 (DP17), 3157.932 (DP18), 3319.503 (DP19), 3482.041 (DP20), 3644.191 (DP21), 3805.332 (DP22), 3967.910 (DP23), 4129.498 (DP24), 4290.457 (DP25), 4453.325 (DP26), 4617.000 (DP27), 4775.306 (DP28). DP represents the degree of polymerization of dextrin chains.

### 2.4. Preparation of Py-Amy

Amylose (350 mg) was dissolved in a 20 mL volume of 1 : 1 mixture of 40 mM aqueous phosphoric acid/DMSO with heating and stirring. After added 1-aminopyrene (10 mg), the system was stirred at 85°C for 45 min under a nitrogen atmosphere and then cooled to room temperature. Then, sodium cyanoborohydride (NaCNBH_3_) was added to a final concentration of 0.2 M. Subsequently, the mixture was maintained at 85°C for 10 h. The crude was obtained following the procedures including vacuum distillation and precipitation by acetone. The crude product was dissolved in water and dialysed with a semipermeable membrane (MWCO 3500 Da) with stirring under 4°C for 24 h. After dialysis, water was removed by vacuum distillation and the product was dried under nitrogen to give purified Py-Amy. UV/Vis (water): *λ*_max _  = 355, 283, and 242 nm; fluorescence (water): excitation/emission = 360/441 nm. The enzymatic hydrolysis product of Py-Amy was analyzed by Q-TOF MS, and Py-Mal was detected. MS (ESI): *m/z*: 566.2017 ([Py-Mal + Na]^+^), 404.1443 ([Py-Mal-glucose + Na]^+^).

### 2.5. Preparation of Self-Assemblies Py-Dex-SWNTs [18]

0.5 mg of SWNTs was added to 5 mL water, followed by mild sonication (at a pulse cycle; 0.2 seconds on, 0.5 seconds off, and 50 W) for 20 min. The resulting fine suspension of SWNTs was mixed with 20 mg Py-Dex dissolved in a 5 mL volume of 3.5 : 1.5 (v/v) mixture of water/DMSO and subsequently sonicated for 4 min, during which the suspended SWNTs were homogeneously dissolved. The solution was heated to about 80°C and a small part of uncoated SWNTs and amorphous carbon were precipitated out. The stable supernatants were collected and then subjected to ultracentrifugation (10,000 g) for 30 min to separate the encapsulated SWNTs. The precipitate was washed with sufficient warm water (*ca.* 35°C) and centrifuged again. This washing/centrifuging cycle was repeated several times and then dried under nitrogen to give Py-Dex-SWNTs. Py-Dex-SWNTs were characterized by the methods of Raman, TEM, and MALDI-TOF MS.

### 2.6. Fluorescence Quenching

Different weight ratios were achieved by changing the amount of SWNTs and keeping the concentration of Py-Dex unchanged. In a typical test, the operation procedures were conducted as follows. 0.4 mg Py-Dex, proper amount of SWNTs, 100 *μ*L DMSO, and proper amount of water were mixed with sonication to a total volume of 667 *μ*L. After that, the mixture was diluted by water to a total volume of 300 mL and resulting solution A. The solution A was used directly for fluorescence quenching tests.

In the fluorescence recovery tests, the samples were prepared by adding proper amount of CDs to solution A. 0.8 mg SWNTs were used to prepare solution A in these tests.

In the chiral sensing tests, the samples were prepared by adding 50 *μ*M *β*-CD to solution A. 0.8 mg SWNTs were used to prepare solution A in these tests.

## 3. Results and Discussion

### 3.1. Preparation and Characterization of Py-Dex, Py-Amy, and Py-Dex-SWNTs

In this study, Py-Dex, Py-Amy, and Py-Dex-SWNTs self-assemblies were firstly prepared and were adequately identified by ^1^H NMR, UV/Vis, fluorescence spectroscopy, transmission electron microscopy (TEM), matrix-assisted laser desorption/ionization-time of flight mass spectroscopy (MALDI-TOF MS), and Raman spectroscopy. The Py-Dex was synthesized by forming a Schiff base between –CHO of the reducing end of dextrin (ring open form) and –NH_2_ of 1-aminopyrene. The yield of product was 85.2% at last. The UV-vis spectrum (see Figure S1 in Supplementary Material at doi:10.1155/2011/862692.) of Py-Dex showed the absorption peaks at 242, 281, 353 nm, representing the characteristic absorption of pyrene moieties. Supplementary Figure S2 also showed the characteristic maximum emission of pyrene moieties at 441 nm, which was the basis for further fluorescent sensing applications. From the ^1^H NMR data of Py-Dex (see Supplementary Figure S3), we could find strong NMR signals arising from protons of glucose units and weak signals arising from protons of pyrene moieties. This is reasonable because each long glucose chain (the average glucose units number was about 20) only linked with one pyrene ring. Most importantly, convincing evidences of covalent linking between pyrene moieties and dextrin chain could be provided by MALDI-TOF MS data (Figure S4). The *m/z* values from the MS spectrum could represent [Py-Dex+H]^+^ and [Py-Dex + Na]^+^ ions, as well (Figure S5).

The preparation of Py-Amy is almost the same to the synthesis of Py-Dex by using amylose instead of dextrin. Considering the large molecular weight of Py-Amy (*M*_*w*_ > 15000), the product was purified by using dialysis (molecular weight cutoff 3500 Da). The UV-vis and fluorescence emission spectra indicated the existence of pyrene moieties (see Figrue  S1and S2). However, NMR and MALDI-TOF MS were not suitable for the characterization of Py-Amy because of the large molecular weight and very low relative content of pyrene moieties (see Figure S3 and S4). Therefore, an enzymatic method was carried out (see Scheme S1). The long *α*-1,4-dextrose chain was firstly cut off by *α*-amylase through the *α*-1,4-glycoside linkage. After sufficient enzymatic hydrolysis, the product was cleaned up with solid phase extraction (to remove the salt and *α*-amylase) and was analyzed by quadrupole time-of-flight MS (Q-TOF MS) (see Figure S5). The characteristic fragment ions at *m/z* 566.2017, which was corresponding to [Py-Mal + Na]^+^, were detected. The second-order MS (MS/MS) was further carried out to confirm the covalent linking between amylose and pyrene moiety. 

**Figure 1:**
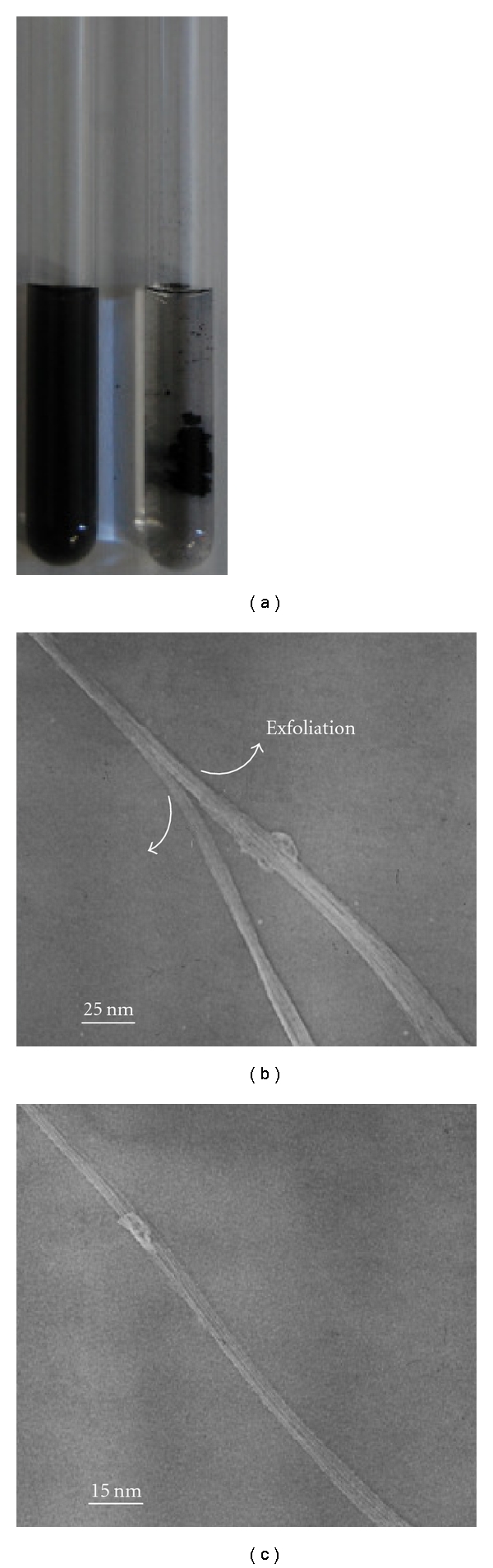
The solubility of SWNTs in water and TEM morphology. (a) Photo of 6 mg/mL Py-Dex-SWNTs (left) and SWNTs in water; (b) and (c) TEM morphology of Py-Dex-SWNTs.

The Py-Dex-SWNTs were prepared by wrapping Py-Dex on SWNTs under proper solution conditions. Furthermore, the dry Py-Dex-SWNTs powder could be obtained through a series of procedures including sonication, boiling, centrifugation, and vacuum drying. This dried powder could be resolubilized in aqueous solution only with mild sonication at room temperature forming homogeneous black solution, implying the well dispersion of SWNTs in aqueous solution (Figure 1(a)). The solubility of Py-Dex-SWNTs in aqueous solution was significant, up to 6 mg/mL. Although dextrin could not be used to solubilize SWNTs (<0.05 mg mL^−1^) [13], Py-Dex was found to be good at solubilizing SWNTs. The remarkable solubilizing capability of Py-Dex could be ascribed to synergistic effect of pyrene moieties and dextrin chain. The *π*-stacking between SWNTs and pyrene rings can help to exfoliate the SWNTs bundles [14]. A clear indication of the exfoliation of the SWNTs bundles was provided by TEM (Figures 1(b) and 1(c)). 

**Figure 2:**
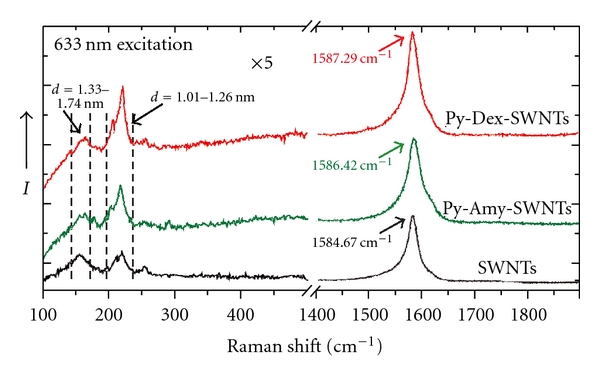
Raman spectra of SWNTs, Py-Dex-SWNTs, and Py-Amy-SWNTs.

Raman spectroscopic measurements were used to confirm the intact SWNT structures in Py-Dex-SWNTs and the interactions between Py-Dex and SWNTs (Figure 2). As a reference, we prepared a Py-Amy-SWNTs composite according to Kim's method [18]. SWNTs possess characteristic Raman peaks at around 190 and 1590 cm^−1^, namely, radial breathing mode (RBM) and tangential G-band, respectively [19]. Comparing the spectrum of pristine SWNTs, slight upshift at G-band position can be observed in that of Py-Dex-SWNTs. Such upshift should be a result of noncovalent assembly by Py-Dex or Py-Amy [20, 21]. The wrapping of Py-Dex on the surface of the SWNTs changed the elastic constant of Py-Dex-SWNTs compared to pristine SWNTs [22]. Further, as indicated in RBM position, the diameter distribution of SWNTs was changed after assembling. The relative content of SWNTs with diameters 1.01–1.26 nm was improved in Py-Dex-SWNTs. The diameter of SWNTs was calculated according to the frequency in RBM [19]. This phenomenon indicated that Py-Dex preferred assembling with smaller tubes.

### 3.2. Tunable Tethering Effect of Dextrin Chains

**Figure 3:**
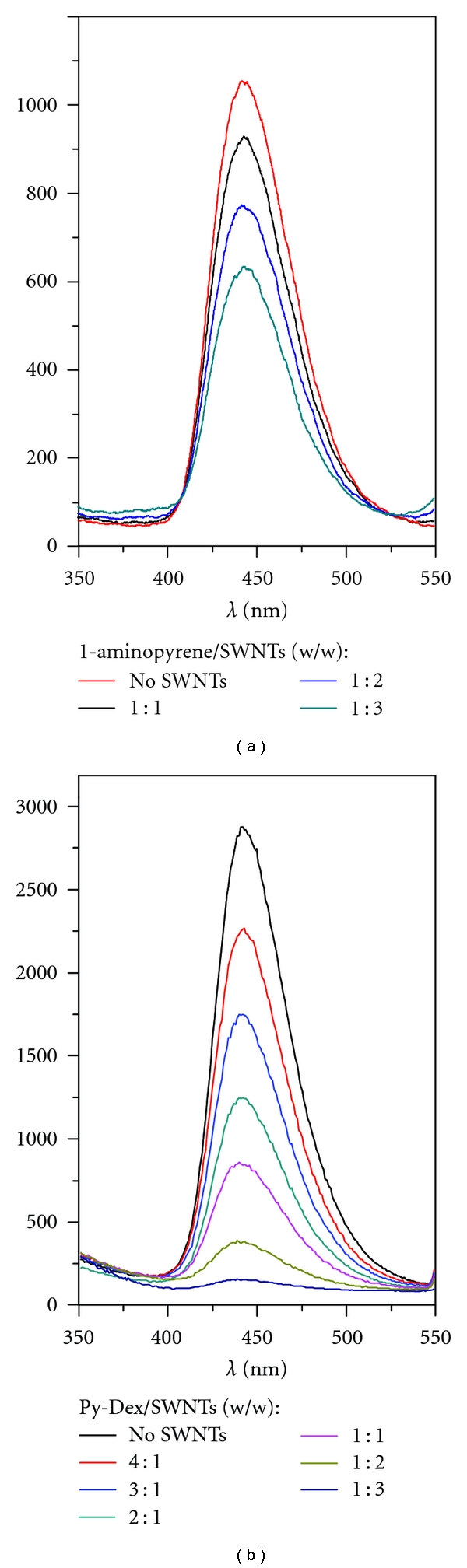
Fluorescence quenching of 1-aminopyrene (a) and Py-Dex (b) by SWNTs under different weight ratios. The concentration of 1-aminopyrene in all the test solutions was kept at 0.22 *μ*M. The concentration of Py-Dex was 1.0 mg/mL. The spectra were all measured with the excitation of 360 nm.

Mostly, the fluorescence of pyrene rings can be quenched by attaching to SWNTs through energy transfer [23]. To elucidate the tethering, especially tunable tethering effect of dextrin chains to pyrene moieties, the fluorescence quenching test was firstly carried out (Figure 3). The quenching was attributed to the increased attachment, which led to a more frequent energy transfer. The details of fluorescence test were presented in the Experimental Section. The fluorescence quenching of 1-aminopyrene, Py-Dex, and Py-Amy with different amounts of SWNTs was compared. First, the fluorescence quenching of 1-amonopyrene by SWNTS was investigated. Stirring was required for preventing precipitation of SWNTs. As shown in Figure 3(a), the fluorescence quenching effect of SWNTs to 1-aminopyrene in aqueous solution was poor. The fluorescence quenching rate ((*I*_0_ − *I*)/*I*_0_) was only about 10% with the coexistence of the same amount of SWNTs (1-aminopyrene: SWNTs, 1 : 1), and it was about 50% when 3 times of SWNTs were added. The poor fluorescence quenching rate could be ascribed to kinetic adsorption of 1-aminopyrene onto SWNTs [23], and thus the attachment of pyrene moieties to SWNTs is unstable and not robust. Secondly, fluorescence quenching of Py-Dex at different weight ratios (Py-Dex:SWNTs, from 4 : 1 to 1 : 3) was compared. As can be seen from Figure 3(b), the fluorescence quenching rate of Py-Dex was about 22% when the weight ratio is 4 : 1, and it was higher than 70% and 95% when the weight ratios were 1 : 1 and 1 : 3, respectively. Obviously, the dextrin chain greatly improved the fluorescence quenching of pyrene moieties by SWNTs. Martin et al. [24] pointed out that shorter distance and tighter attachment between dyes and SWNTs were beneficial to energy transfer quenching. Hence, the results revealed that the dextrin chains indeed played an obvious role in making pyrene moieties more effectively attach to SWNTs allowing more frequent energy transfer from pyrene to SWNTs, and we called this “tethering effect”. 

**Figure 4:**
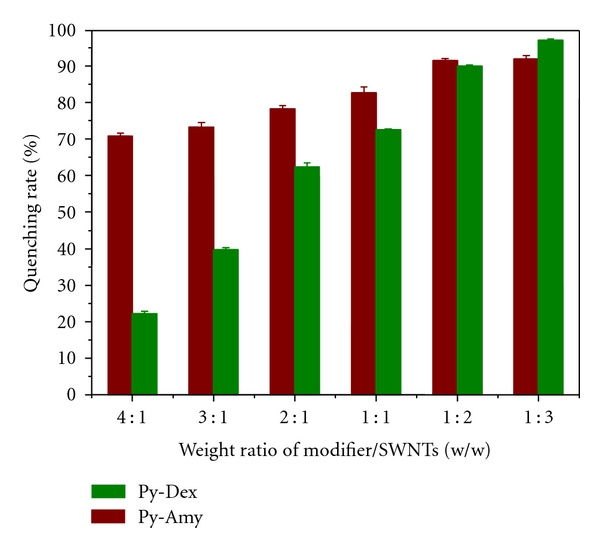
Fluorescence quenching tests of Py-Dex and Py-Amy by different amounts of SWNTs, exited at 360 nm and monitored at 440 nm. The concentration of Py-Dex and Py-Amy in all the test solutions was kept at 1.0 mg/mL.

Finally, the fluorescence quenching of Py-Amy was investigated as a control. As compared in Figure 4, tunable tethering effect of dextrin chains could be drawn. At a same ratio of 4 : 1, fluorescence of pyrene in Py-Amy was quenched up to 70.9% which is distinctly higher than 22.4% for Py-Dex, indicating that longer dextrin chains gave stronger tethering effect. With increasing amount of SWNTs, pyrene moieties in Py-Dex and Py-Amy were both sufficiently tethered to SWNTs and the quenching rates both trended to 100%.

**Figure 5:**
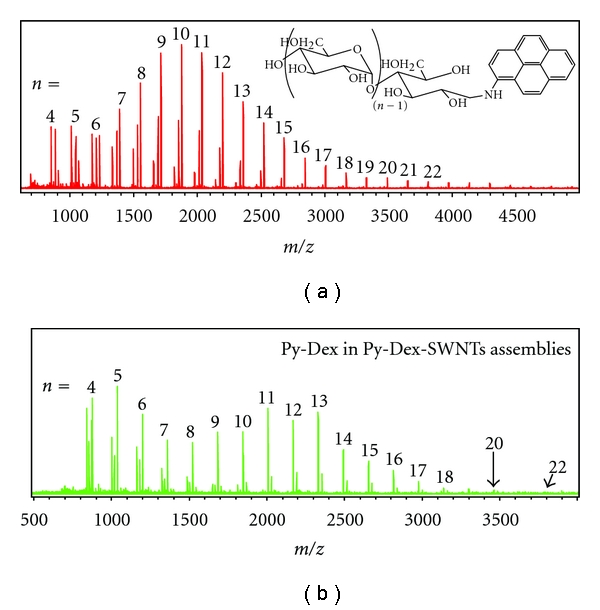
MALDI-TOF MS spectra of (a) Py-Dex and (b) dry powder of Py-Dex-SWNTs assemblies.

A more distinct evidence of tunable tethering was provided by MALDI-TOF MS. According to the results of MALDI-TOF MS, the dextrose chain length (number of dextrose units) distribution of Py-Dex is ranging from 4 to 25 (Figure 5(a)). After assembling with SWNTs, the relative abundance of different components was changed (Figure 5(b)). The components with chain length from 11 to 13 presented the highest relative abundant. Although the components with chain length from 11 to 17 were relatively less in Py-Dex, those components showed obvious concentration in Py-Dex-SWNTs revealing that longer dextrose chains could provide stronger tethering effect. The MS signals of the components with chain length longer than 17 were still relatively weak. This could be ascribed to the more difficult dewrapping of the long chain components from SWNTs. Besides, the difficulty of ionization of the long chain compounds should also contribute to the weak signals. On the other hand, the relative abundance of the components with chain length from 4 to 6 was rather high should it be ascribed to the easier desorption of the short chain components.

**Figure 6:**
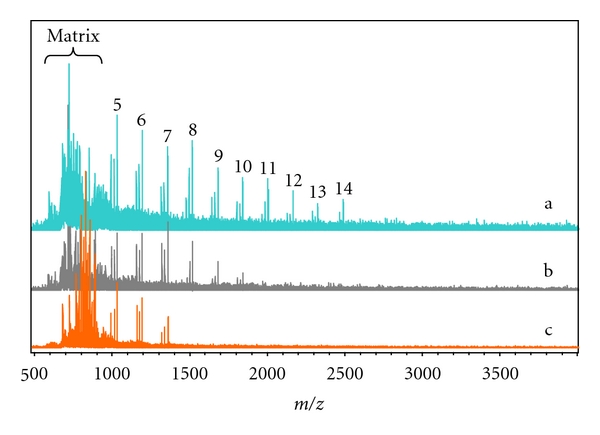
MALDI-TOF MS spectra of centrifugation supernatants of Py-Dex aqueous solutions after assembling with different amounts of SWNTs. Weight ratios of Py-Dex to SWNTs are (a) 4 : 1, (b) 3 : 1, (c) 2 : 1.

The tunable tethering of dextrin chains was further investigated from another viewpoint. The assembling of Py-Dex and SWNTs was carried out with different weight ratios from 4 : 1 to 2 : 1 in aqueous solutions. After assembling, each solution was centrifuged (10,000 g) and the residues (free components that did not tether to SWNTs) in the supernatants were investigated by MALDI-TOF MS (Figure 6). Because of competitive wrapping, components with longer chains preferentially disappeared in the supernatants, showing gradually increase of tethering effect as the increase of chain length.

Hence, we could control the tethering effect of dextrin chains to the pyrene moieties by adjusting the length of dextrin chains. Narrow or even single molecular weight distributed dextrin could be readily prepared from the products of starch hydrolysis by preparative size-exclusion chromatography [25]. So, the narrow distributed dextrin could be used to precisely control the strength of tethering, and the fluorescence of the self-assemblies can be fine tuned without changing their solubility. Such self-assemblies are potential for the applications in molecular recognitions. In addition, tunable tethering of other target molecules to SWNTs for different purpose should be interesting, as well.

### 3.3. Chiral Selective Sensing

**Figure 7:**
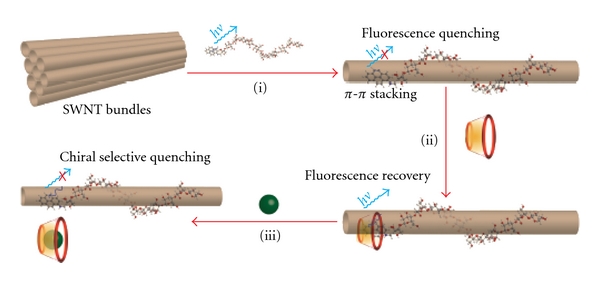
Schematic illustration of the principle of chiral selective sensing. Dispersion of SWNTs and fluorescence quenching of pyrene moieties (step i); fluorescence recovery of pyrene moieties through hydrophobic inclusion by CDs (step ii); chiral selective fluorescence quenching through competitive occupation of CD cavities by enantiomers (step iii).

Inspired by the tethering effect of dextrin chains toward pyrene moieties, we further explored applications in rapid chiral sensing. CDs was introduced to the Py-Dex-SWNTs assemblies as chiral binding sites. As shown in Figure 7, chiral sensing would be achieved by three steps: first, the dispersion of SWNTs and fluorescence quenching of pyrene moieties (step i); secondly, fluorescence recovery of pyrene moieties through hydrophobic inclusion by CDs (step ii) and thirdly, chiral selective fluorescence quenching through competitive occupation of CD cavities by enantiomers (step iii). In such a solution, the SWNTs were individually dispersed and the fluorescence of pyrene moieties were sufficiently quenched (quenching rate >90%) by SWNTs with the support of dextrin chains.

**Figure 8:**
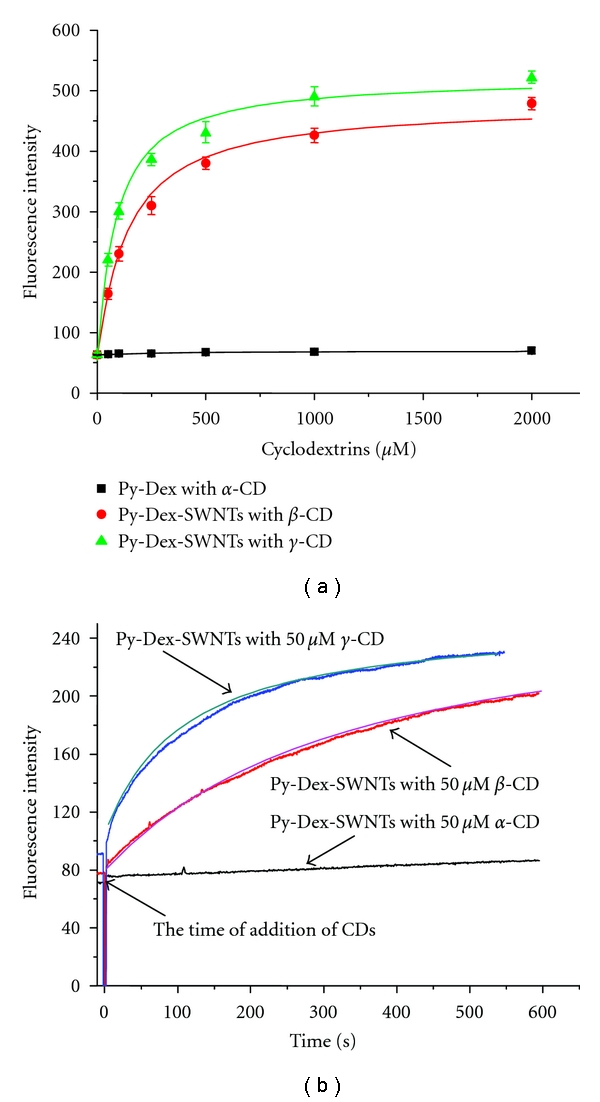
Fluorescence recovery effect of *α*-, *β*-, and *γ*-CDs on Py-Dex-SWNTs systems. Fluorescence was excited at 360 nm and monitored at 440 nm. Testing operations and other conditions were given in Section 2.

As proved by in [26], the hydrophobic cavities of CDs were able to cap the pyrene rings through hydrophobic inclusion. Since the capping of CD cavities, the tight attachment between pyrene and SWNTs would be broken resulting in the recovery of fluorescence. The *α*-, *β*-, and *γ*-CD were chosen to verify this design. The results displayed that both *β*- and *γ*-CD obviously recovered the fluorescence of Py-Dex-SWNTs system except *α*-CD (Figure 8(a)). Simultaneously, the kinetic of the fluorescence recovery was investigated. As shown in Figure 8(b), the fluorescence reached a plain for about 10 min by *β*- or *γ*-CDs. The kinetic of fluorescence recovery was not slow and would not affect the rapid applications, because we could prepare a stock solution before further tests. Besides, the fluorescence did not change by *α*-CD. The results should be attributed to the proper size of the cavities of *β*- and *γ*-CD [27] and limited size of the cavity of *α*-CD [28] for capping pyrene moieties.

**Figure 9:**
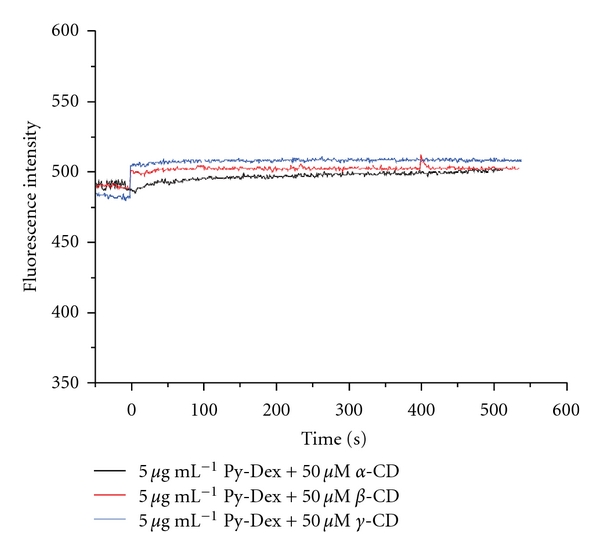
Time-dependent emission fluorescence of Py-Dex upon addition of CDs. Fluorescence was excited at 360 nm and monitored at 440 nm.

In order to further prove the mechanism of fluorescence recovery, the time-dependent fluorescence feature of Py-Dex (without SWNTs) was monitored upon the adding of *α*-, *β*-, and *γ*-CDs. As shown in Figure 9, the fluorescence kept constant within 550 s upon addition of each kind of CDs. This meant that the existence of SWNTs was necessary for the control of fluorescence intensity and the capping effect of CD cavities was the motivity of fluorescence recovery. 

**Figure 10:**
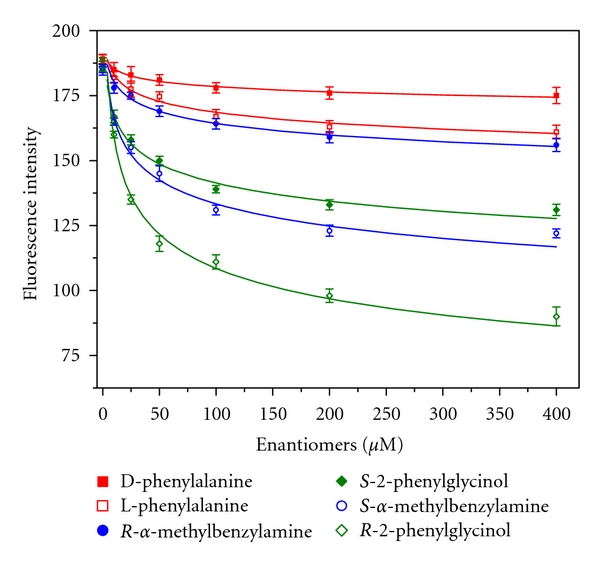
Chiral selective sensing of three pairs of enantiomers. Testing operations and other conditions were given in Section 2.

Subsequently, the triad Py-Dex-SWNTs/*β*-CD system was applied to chiral selective sensing. As illustrated in Figure 7 (step iii), the enantiomers could extrude the pyrene moieties out of the CD cavities to a certain percent through competitive occupation. Consequencely, pyrene moieties could effectively reattach to the surface of SWNTs, because they were confined around the SWNTs by dextrin chains all through. Owing to the chirality of CD cavities, the interaction constant between CDs and enantiomers would be different and thus the competitive occupation would also be different. Therefore, the chiral selective fluorescence quenching could be achieved.  Figure 10 showed the successful chiral recognition of three pairs of enantiomers. For *α*-methylbenzylamine enantiomers, the fluorescence of 1-SWNT/*β*-CD system was more obviously quenched by *S*-isomer than *R*-isomer, indicating the stronger interaction between *S*-isomer and *β*-CD. This was in well accordance with the thermodynamic results [29]. Similar results were observed upon analyzing phenylalanine enantiomers. L-phenylalanine exhibited stronger interaction with *β*-CD cavities, which was also supported by the reported results [30].

The normalized ratios of the quenched fluorescence intensity, that was Δ*I*_*R*_/Δ*I*_*S*_ or Δ*I*_*S*_/Δ*I*_*R*_, were used to evaluate the chiral selectivity of the present method. Here, Δ*I* = *I*_0_ − *I*, *I*_0_, and *I* were fluorescence intensity of 1-SWNT/*β*-CD system before and after the addition of enantiomers, respectively. As can be seen from Table 1, chiral selectivity larger than Py-Amy was obtained. The results indicated that the chiral selectivity of present method was comparable with reported chiral sensors [31, 32]. 

Moreover, employment of other chiral selectors such as crown ethers, calixarenes, and various CD derivatives would make the present method applicable to a wider range of enantiomers.

## 4. Conclusion

In summary, self-assemblies of SWNTs through tunable tethering of pyrenes by dextrin chains were prepared. The pyrene-grafted dextrin Py-Dex was obtained by covalently linking pyrene to the reducing end of dextrin chain. Because of the synergistic effect of dextrin chains and pyrene moieties, the Py-Dex-SWNTs assemblies were significantly water-soluble, and the homogeneous black solutions of Py-Dex-SWNTs have been shown to be as stable for more than two weeks. It was found out that dextrin chains had a tethering effect toward the pyrene moieties on the surface of SWNTs. Moreover, the tethering effect was tunable by adjusting the length of dextrin chains. The fluorescence of pyrene moieties was sufficiently quenched by SWNTs with the support of dextrin chains. In chiral selective sensing experiments, CDs were introduced into the Py-Dex-SWNTs system as chiral binding site. The inclusion of CD cavities to pyrene could recover the quenched fluorescence, and a chiral recognition method was established based on fluorescence sensing. Through competitive occupation of the enantiomers to CD cavities, the fluorescence was quenched chiral differently. In particular, a rapid chiral sensing platform using triad Py-Dex-SWNTs/*β*-CD system was successfully fabricated. The results were of significance for the potential applications of self-assemblies of SWNTs in molecular recognition researches.

##  Supporting Information Available

Details on the following topics are available: UV/Vis spectra of Py-Dex, Py-Amy, and 1-aminopyrene; Fluorescence emission spectra of Py-Dex, Py-Amy, and 1-aminopyrene; ^1^H NMR spectrum (600 MHz, in DMSO-D6) of Py-Dex; MALDI-TOF MS spectrum of Py-Dex; Q-TOF MS of the enzymatic solution of Py-Amy. Enzymatic method for the characterization of Py-Amy.

## Figures and Tables

**Table 1:** Chiral selectivity of the present method for three pairs of enantiomers under different concentration levels.

Concentration of Enantiomer (*μ*M)	10	50	100	400
Phenylglycinol	1.35	1.58	1.78	1.80
Methylbenzylamine	2.17	2.50	2.59	2.59
Phenylalanine	1.75	1.80	2.00	2.00
